# Structure-Function Relationship of Substituted Bromomethylcoumarins in Nucleoside Specificity of RNA Alkylation

**DOI:** 10.1371/journal.pone.0067945

**Published:** 2013-07-03

**Authors:** Stefanie Kellner, Laura Bettina Kollar, Antonia Ochel, Manjunath Ghate, Mark Helm

**Affiliations:** 1 Institute of Pharmacy and Biochemistry, Johannes Gutenberg University Mainz, Mainz, Germany; 2 Institute of Pharmacy, Nirma University, Ahmedabad, Gujarat, India; Ben-Gurion University of the Negev, Israel

## Abstract

Selective alkylation of RNA nucleotides is an important field of RNA biochemistry, e.g. in applications of fluorescent labeling or in structural probing experiments, yet detailed structure-function studies of labeling agents are rare. Here, bromomethylcoumarins as reactive compounds for fluorescent labeling of RNA are developed as an attractive scaffold on which electronic properties can be modulated by varying the substituents. Six different 4-bromomethyl-coumarins of various substitution patterns were tested for nucleotide specificity of RNA alkylation using tRNA from *Escherichia coli* as substrate. Using semi-quantitative LC-MS/MS analysis, reactions at mildly acidic and slightly alkaline pH were compared. For all tested compounds, coumarin conjugates with 4-thiouridine, pseudouridine, guanosine, and uridine were identified, with the latter largely dominating. This data set shows that selectivity of ribonucleotide alkylation depends on the substitution pattern of the reactive dye, and even more strongly on the modulation of the reaction conditions. The latter should be therefore carefully optimized when striving to achieve selectivity. Interestingly, the highest selectivity for labeling of a modified nucleoside, namely of 4-thiouridine, was achieved with a compound whose selectivity was somewhat less dependent on reaction conditions than the other compounds. In summary, bromomethylcoumarin derivatives are a highly interesting class of compounds, since their selectivity for 4-thiouridine can be efficiently tuned by variation of substitution pattern and reaction conditions.

## Introduction

### RNA labeling

Scientific investigations of the principle biopolymers face a need for effective and selective labeling agents. This applies in particular to ribonucleic acids (RNA), which have such divergent functions as transient information keepers, adaptor molecules for the genetic code, scaffold and catalytic center in protein biosynthesis, and versatile regulators of gene expression. Labeling is a prerequisite for various experimental approaches in RNA research. Commonly applied labeling procedures for RNA synthesized *in vitro* can be classified according to whether they are conducted during or after enzymatic [1] or synthetic [2–5] RNA synthesis, thus being referred to as co-transcriptional, or co-synthetic labeling in the former case, and as post-transcriptional or post-synthetic labeling in the latter [6–8]. A hybrid strategy includes the co-synthetic introduction of a functional group instead of the actual label, and a second post-synthetic step during which the functional group may be selectively conjugated to a reactive dye [9]. This strategy has recently been adapted to RNA synthesized in living cells, e.g. by feeding cells with analogues of conventional nucleosides, such as 5-ethinyluridine (5EU) [10] or 4-thiouridine (s^4^U) [11]. The analogues are incorporated into nascent RNA by the cellular transcription machinery, and can subsequently be post-synthetically labeled. In all post-labeling reactions, the selectivity of the reactive dye for a particular unique functional group in the RNA is of paramount importance. The success of e.g. 5EU is largely based on the extreme specificity of its Cupper (I) dependent azide-alkyne cylcloaddition (CuAAC) conjugation to azide derivatives of various labels [10]. The selectivity of the CuAAC reaction is such, that virtually no side reactions occur with any functional group present in biological material, and the reaction is thus called bioorthogonal [12]. For native RNA isolated from biological material, introduction of functional groups that may potentially be used for site specific labeling does actually occur *in vivo*. More than 100 chemically distinct post-transcriptional modifications have been found in native RNA, and a number of them has been explored for site-specific labeling already [7,13–18].

### Labeling agents

Among the available labeling agents, fluorescent labels predominate. In so called reactive dyes, a reactive functional group is appended to the fluorescent moiety itself. In addition to azides [10] and terminal alkynes [19] for click labeling, nucleophiles like thiols [20], primary amines [21], and hydrazones [22] are in use. One particular class of reactive compounds of interest are electrophiles such as NHS-esters [8], isothiocyanates [21], and alkylhalides [23]. Alkylation and acylation target nucleophilic sites in RNA, whose reactivity is well characterized. Early on, treatment of nucleic acids with electrophiles was mostly aimed at the deduction of structural features and at understanding the carcinogenic features of alkylating agents [24]. Overall, the most reactive electrophiles such as alkylnitrosourea were found to alkylate all oxygens and nitrogens in nucleic acids [25], whereas a host of more moderately reactive electrophilic agents typically target nitrogens with various degrees of selectivity [26]. After Maxam & Gilbert type sequencing [27] with electrophiles was driven back by Sanger sequencing [28], the development of new electrophiles with pronounced selectivity slowed down, until recently SHAPE sequencing was developed, with reagents exquisitely selective for the 2’oxygen [29]. Combination with reverse transcription techniques [30] and, ultimately, RNA Seq techniques, has now boosted transcriptome wide structural probing [31–33].

### Selectivity of electrophilic labeling agents

Specific targeting of non-canonical nucleotides with reactive dyes depends on the selectivity of the reactive dye for a particular modification *versus* other functional groups present in canonical RNA nucleotides, e.g. exocyclic amines. Examples for selectively targeted nucleophilic RNA modifications include primary amines [34], pseudouridines [14–17], thiouridine [35] and a few others [7]. However, a reagent exposing “perfect” selectivity akin to orthogonality, as measured by the CuAAC gold standard, has not been characterized.

**Figure 1:**
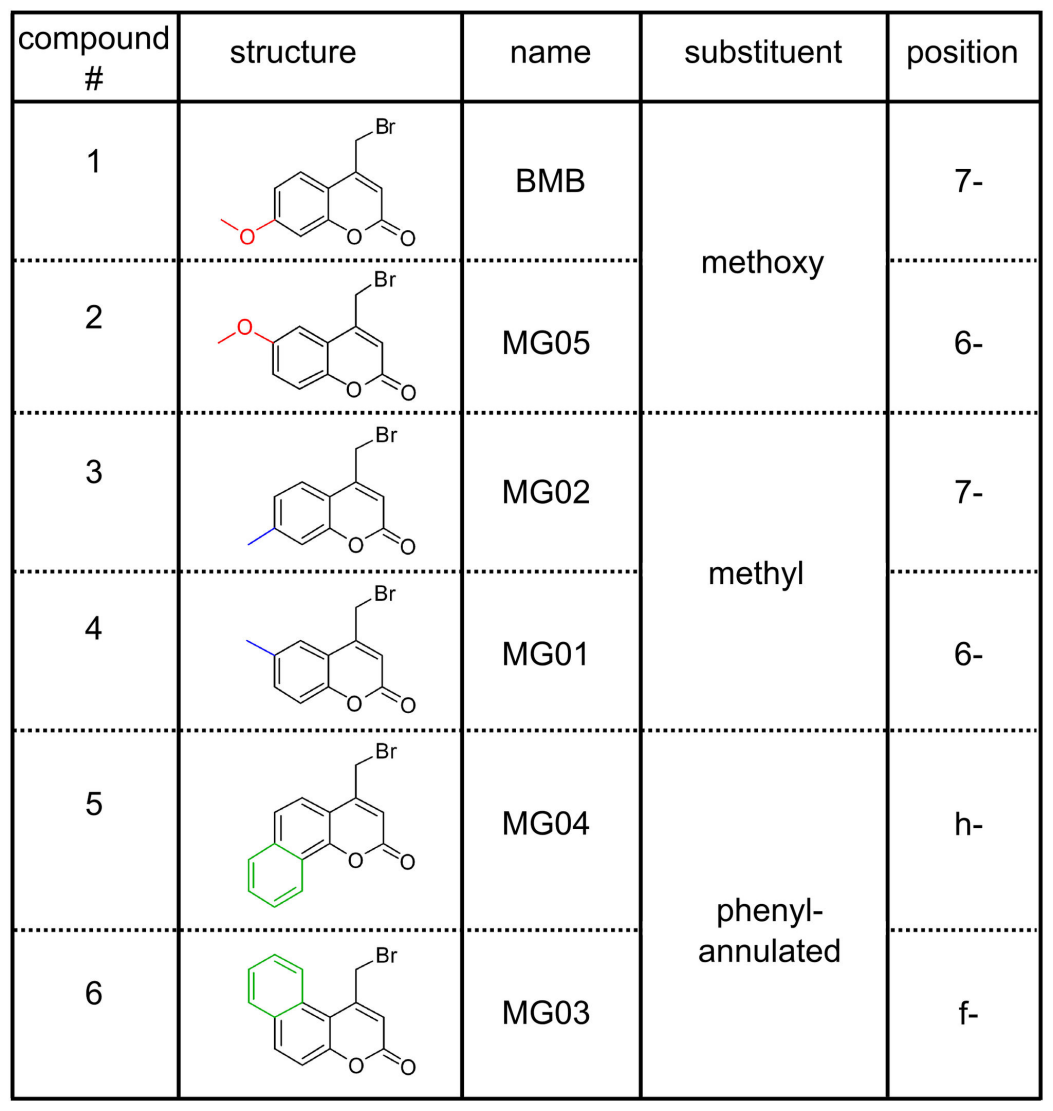
Coumarins used in this study. The coumarins are isomeric pairs, substituted with 3 different substituents (color code: methoxy-red, methyl-blue, phenyl-annulated green).

While screening the literature for pairs of RNA modifications and corresponding labeling agents [7], we found only one example for a coalescence of the reactive electrophile and a fluorescent dye into a single scaffold, as opposed to just linking the two moieties via a series of covalent bonds. Most interestingly, this example concerned bromomethylcoumarines, in particular 4-bromomethyl-7-methoxycoumarin (BMB) (see Figure 1 first panel). BMB was reported to selectively alkylate the uridine derivatives pseudouridine (Ψ) and 4-thiouridine in reactions with native tRNA [36]. Furthermore, the reaction conditions were reported to influence the selectivity to a significant extend, including selective alkylation of pseudouridine. Compared to various aforementioned, relatively small alkylating agents, the coumarin scaffold has the advantage of being directly detectable due to its fluorescent properties, and to allow incorporation of additional functional groups e.g. for further functionalization. Therefore, we have recently made use of the coumarin scaffold and introduced an azide function at position 7, in order to study alkylation specificity of the resulting compound termed N3BC [37]. In our hands, N3BC displayed selectivity for uridine over the other major ribonucleotides, but not for pseudouridine. N3BC contains an electron withdrawing azide substituent where the presumed Ψ-selective BMB contains a methoxy-function, whose +M-effect is known to increase electron density in the aromatic system. This raised the possibility that the specificity of bromomethylcoumarins in RNA alkylation may be modulated by the coumarin substitution pattern. During a literature survey of selective alkylating agents we noticed a flagrant underrepresentation of studies employing a basic principle well developed on other areas of bioorganic and medicinal chemistry, namely structure-function relationship by variation of the active small molecule (compare e.g. [38]).

We therefore decided to validate the suitability of bromomethylcoumarins as a study object in structure-function relationships of RNA alkylation whose electronic properties can be tuned by varying the substituents. We have now re-examined BMB in addition to 5 other coumarin derivatives, which are shown in Figure 1, with total tRNA *Escherichia coli* (*E. coli*). In this study we discuss the differences in alkylation efficiency depending on the position and the character of the substituent and how buffer conditions influence the selectivity for certain nucleotides.

## Materials & Methods

### Coumarins used in this study

4-Bromomethyl-7-methoxycoumarin (BMB) was purchased from Sigma-Aldrich (Munich, Germany). Compounds **2** to **6** were synthesized from different substituted phenols treated with ethyl-4-bromoacetoacetate. The ethyl-4-bromoacetoacetate was obtained by bromination of ethylacetoacetate [39]. Ethyl-4-bromoacetoacetate was then treated with 4-methoxy phenol, 3-cresol, 4-cresol, 1-napthol and 2-napthol under Pechmann cyclisation condition using concentrated sulphuric acid to afford the differentially substituted 4-bromomethyl coumarins (2–6), respectively [40,41]. All coumarins were dissolved in pure DMSO to give a 20 mM solution.

### Reaction of coumarins with RNA

#### RNA used in this study

Total tRNA *E. coli* was prepared by gel-purification of total RNA from *E. coli* (Roche, Mannheim, Germany)

*In-vitro-*transcript of *Mus musculus* (*M. m.*) tRNA^Asp^ was prepared by *in vitro* transcription as described by Jurkowski et al. [42] and gel-purified.

The quality of the used tRNA was analyzed on a 10% denaturing urea polyacrylamide gel. The tRNA bands were stained with GelRed (Biotium, Hayward, CA, USA) and imaged on a Typhoon (GE Healthcare) with excitation at 532 nm and emission filter 610 nm. For IVT, only one major band can be seen. The native tRNA shows two tRNA bands (70 and 85 nt), as expected (see Figure S1 in File S1).

#### Derivatization conditions 1

0.5 µg/µL tRNA (16.67 µL of 3 µg/µl tRNA stock solution) was incubated with 8.8 mM coumarin (44.13 µL of stock at 20 mM dissolved in pure DMSO), 62.5 mM phosphate buffer (6.25 µL of 1 M stock pH 6.5) and 75% DMSO (30.88 µL pure DMSO and 2.1 µL water) at 37 °C for 300 minutes under light protection. After the reaction, 10 volumes of a 2% LiClO_4_ in aceton solution were added and RNA was precipitated by centrifugation at 15000 g at room temperature for 30 minutes and the pellet washed with pure aceton. The RNA was redissolved in pure water to yield a 0.5 µg/µL solution.

#### Derivatization conditions 2

0.5 µg/µL tRNA (16.67 µL of 3 µg/µl tRNA stock solution) was incubated with 10 mM coumarin (50 µL of stock at 20 mM dissolved in pure DMSO), 100 mM phosphate buffer (10 µL of 1 M stock pH 8.25) and 70% DMSO (20 µL pure DMSO and 3.23 µL water) at 37 °C for 180 minutes under light protection. The tRNA workup was the same as described for conditions 1.

#### PAGE analysis of coumarin treated tRNA

The concentration of coumarin-conjugated tRNA was determined using a Nanodrop-ND-2000 (Peqlab, Erlangen, Germany) and 50 µg were analyzed on a 10% urea gel. Blue fluorescence of coumarin labeled tRNA was observed upon radiation with UV light (365 nm) and imaged with a Geldoc (Peqlab, Erlangen, Germany). Afterwards, the tRNA was stained for 10 minutes with GelRed (Biotium, Hayward, CA, USA).

### LC-MS method and analysis

#### Sample preparation

10 µg of coumarin treated tRNA (final concentration 1 µg/µL) was digested with Nuclease P1, Snake Venom Phosphodiesterase and Shrimp Alkaline Phosphatase, as previously described [37].

#### LC-MS and LC-MS/MS analysis

The digested RNA was analyzed on an Agilent 1260 series equipped with a diode array detector (DAD) and Triple Quadrupole mass spectrometer Agilent 6460. A Synergy Fusion RP column (4 µm particle size, 80 Å pore size, 250 mm length, 2 mm inner diameter) from Phenomenex (Aschaffenburg, Germany) was used at 35 °C. The solvents consisted of 5 mM ammonium acetate buffer adjusted to pH 5.3 using acetic acid (solvent A) and pure acetonitrile (solvent B). The elution started with 100% solvent A for 2 minutes followed by a linear gradient to 20% solvent B at 10 min. For complete coumarin-conjugate elution solvent B was increased to 50% at 15 minutes. Initial conditions were regenerated by rinsing with 100% solvent A for 8 minutes. The flow rate was 0.5 mL/min.

**Figure 2:**
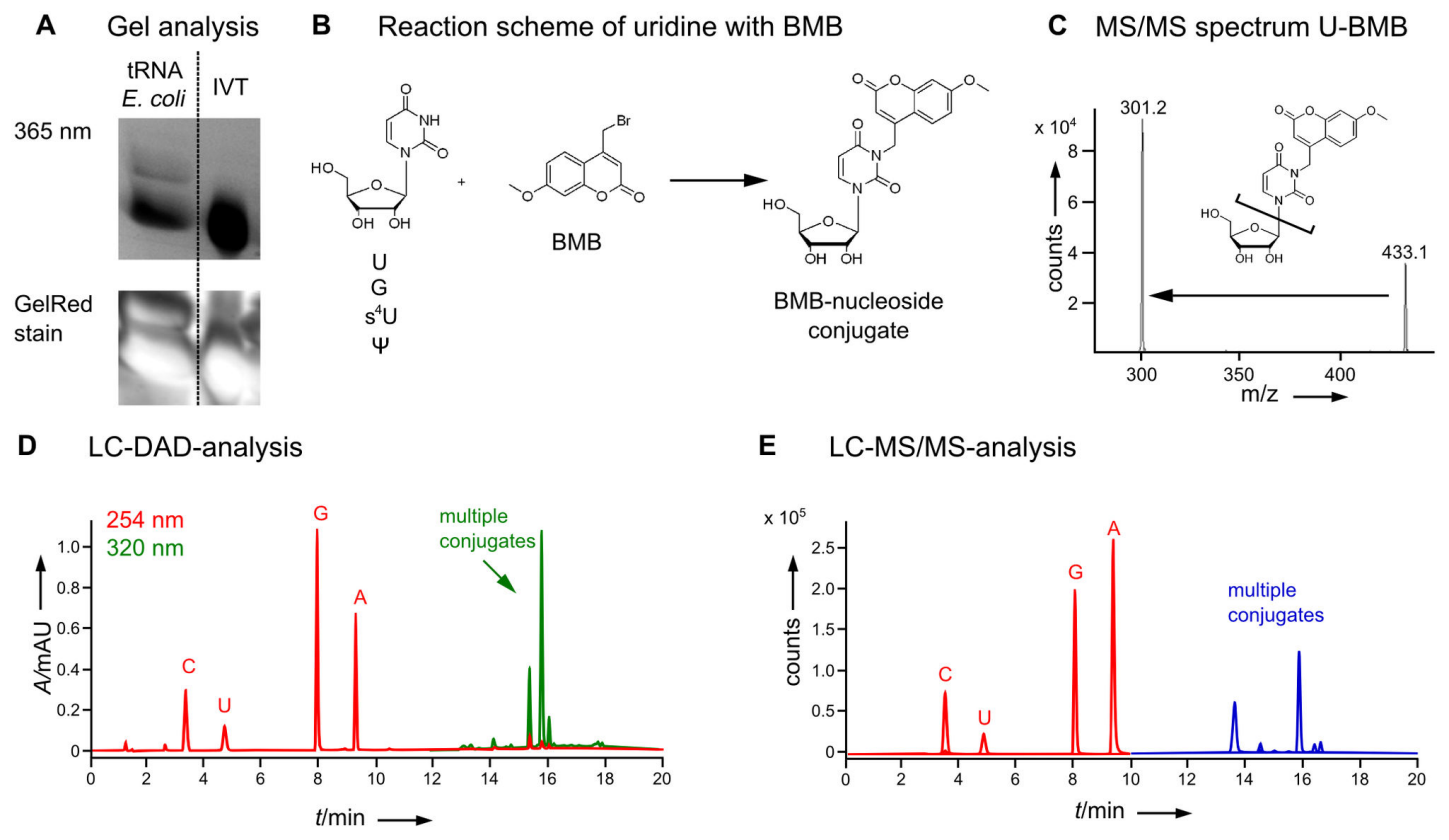
Reaction of BMB with tRNA following the reaction conditions described by Yang & Soell [36]. A) In-gel detection of tRNA-BMB-conjugates of total tRNA from *E. coli* and in-vitro-transcript (IVT) tRNA in a polyacrylamide gel. The fluorescence was imaged upon excitation at 365 nm with a GelDoc and the staining control with GelRed was imaged on a Typhoon. B) Possible reaction mechanism of BMB with uridine as an exemplary nucleoside. C) Mass spectrum, structure and main fragmentation of positively charged [M+H]^+^ of BMB-uridine-conjugate. The Mass transition used in (E) is indicated by an arrow. D) HPLC analysis of total tRNA *E. coli* reacted with BMB, digested to nucleosides and detected with a diode array detector (DAD). The red chromatogram shows nucleoside absorption at 254 nm and the green chromatogram absorption at 320 nm of BMB and its conjugates. Peaks overlapping in both chromatograms indicate possible BMB-nucleoside conjugates. E) LC-MS/MS analysis of total tRNA *E. coli* reacted with BMB and digested to nucleosides using the mass transitions given in Table S1 in File S1.

The effluent from the column was first measured photometrical at 254 nm and 320 nm by the DAD, followed by the mass spectrometer equipped with an electrospray ion source (Agilent Jet Stream). ESI parameters were as follows: gas temperature 300 °C, gas flow 5 L/min, nebulizer pressure 35 psi, sheath gas temperature 350 °C, sheath gas flow 12 L/min and capillary voltage 3500 V. The MS was setup in the MS2scan mode to scan a mass range of 50 to 1000 Dalton in positive ion mode for coumarin-conjugate identification. 10 µg of conjugated tRNA *E. coli* were injected and the masses coinciding with UV-signals at 320 nm were used for detailed analysis in a second sample injection in the product ion scan mode. Therefore quadrupole 1 was adjusted to filter the detected masses, followed by fragmentation at 15 eV collision energy in the collision cell and final mass fragment analysis in quadrupole 2. One of the resulting mass spectra is shown in Figure 2C. The resulting mass-transitions for BMB can be found in Table S1 in File S1 and for the other coumarin-conjugates in Table S2-S6 in File S1.

### Relative quantification of coumarin conjugates

As detailed in the results section, assessment of relative amounts of coumarin conjugates requires three normalization steps, to account for (i) the injected amount of sample (n_A_ ; normalization to adenosine peak as internal standard); (ii) differential detection efficiency by MS (r_f_ ; response factor); and (iii) the relative abundance of nucleosides in the starting material RNA preparation (c_ra_ ; correction for relative abundance).

**Figure 3:**
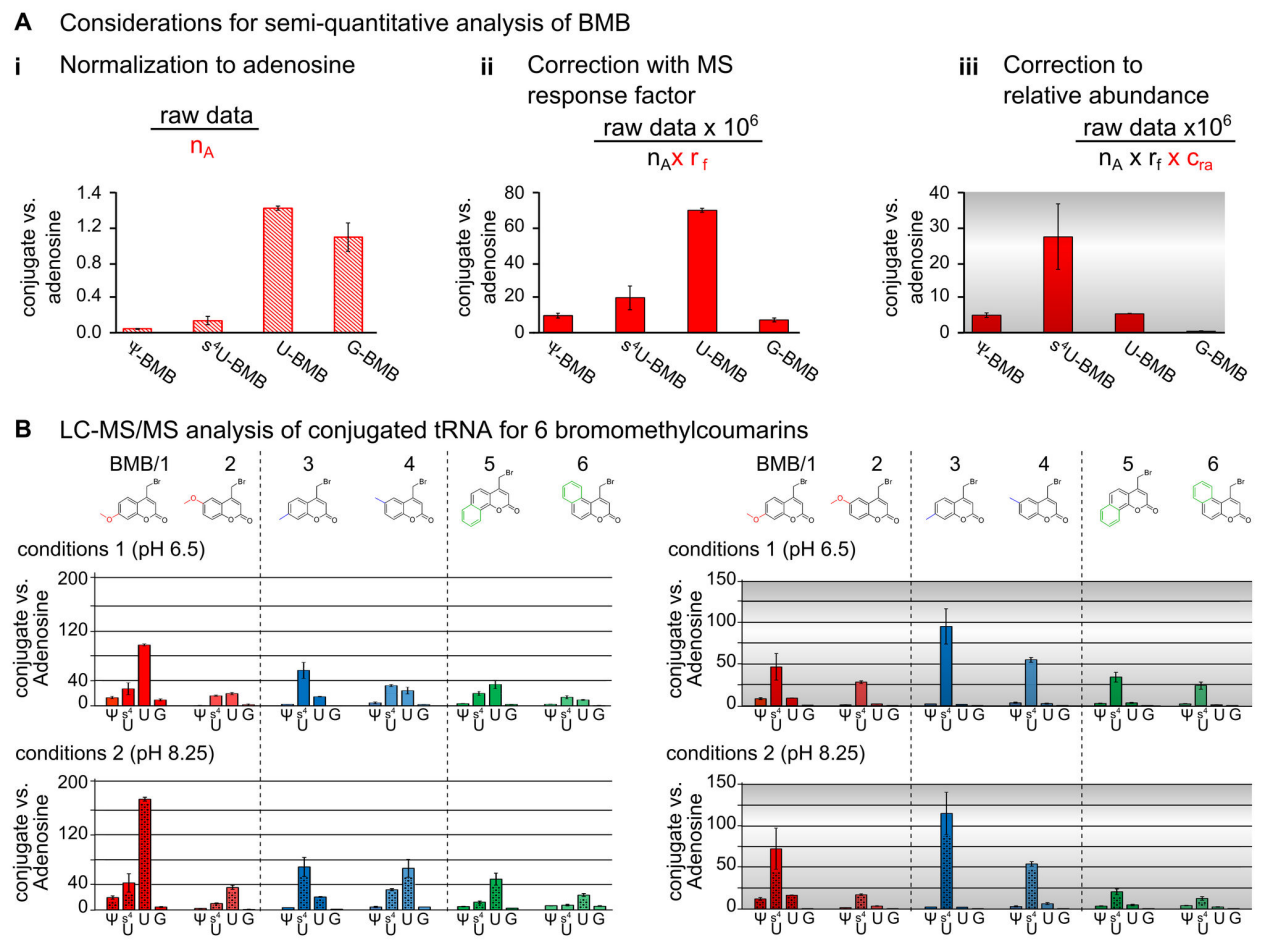
Data analysis of LC-MS/MS experiments of total tRNA *E. coli* treated with bromomethylcoumarins. **A**) LC-MS/MS results from the chromatogram in Figure 2E after peak integration and data processing. The influence of the raw data processing steps is demonstrated for BMB conjugates. (i) Normalization to adenosine (n_A_) for intersample comparability reveals guanosine and uridine as the main reaction partners of BMB (ii). Usage of the found response factors (r_f_) which account for the differential ionization efficiencies in the mass spectrometer indicates U-BMB has the main reaction product. Data processing using n_A_ and r_f_ are used to display the reactivity of BMB (iii). Normalization to the relative abundance of tRNA modifications with the correction factor c_ra_. This last data processing step is used to assess the selectivity of BMB for the substrate nucleosides. Here, 4-thiouridine is the main reaction partner with BMB.B) Reaction of BMB with total tRNA *E. coli* in comparison to 5 coumarins with different substitution patterns. On the top the chemical structure of all used coumarins are shown. The diagram below shows LC-MS/MS results of all coumarin-nucleoside conjugates from total tRNA *E. coli* reaction digests under slightly acidic conditions 1 (pH 6.5) monitored by their respective mass transitions (see Table S1-S6 in File S1) using the normalization to adenosine (factor n_A_). For comparison the mass integrals were corrected by usage of a response factor (r_f_) derived from absorption at 320 nm. The colors of the bars fit to the colors of the coumarin structures above. The graph displays the results for the reaction under more alkaline conditions 2 (pH 8.25), using the same data processing. Under these conditions the reactivity of guanosine with the coumarins is decreased and uridine is the prominent reaction partner. C) Reaction of all 6 differently substituted coumarins considering the nucleoside abundance (factor c_ra_) by analysis of tRNA *E. coli* composition. The upper graph is for reaction conditions 1, the graph below for reaction conditions 2. The processed data clearly indicates a preference for 4-thiouridine of all tested coumarins which is most pronounced for reaction conditions 1 compared to conditions 2.

**Figure 4:**
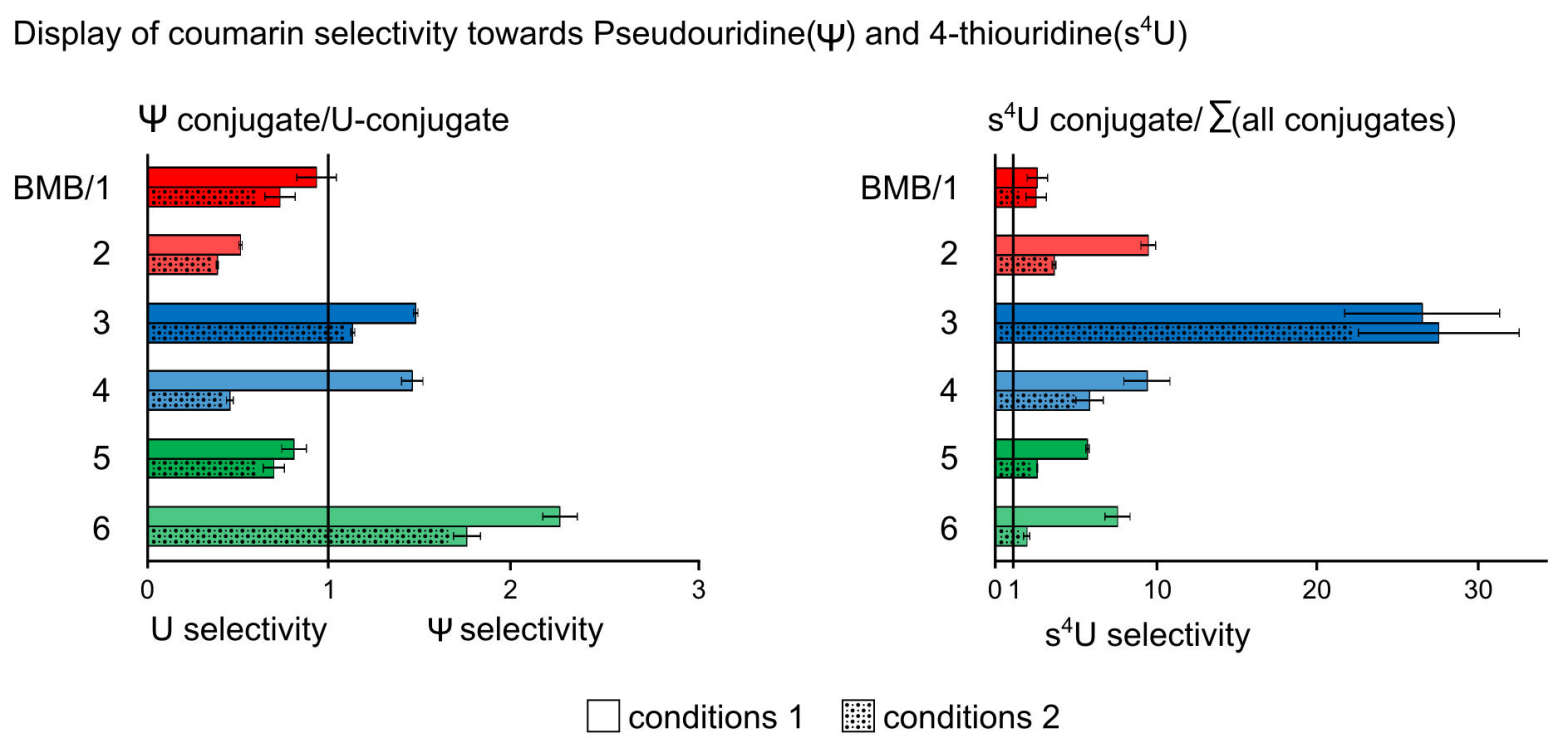
The left diagram shows the nucleoside selectivity calculated for pseudouridine by dividing the U-conjugate abundance by the Ψ-conjugate abundance. All numbers below 1 present selectivity for uridine over pseudouridine and *vice versa* for all numbers above 1. In case of the main conjugates with 4-thiouridine the calculation includes the sum of all conjugates. In all cases conditions 1 lead to the highest overall selectivity for 4-thiouridine except for compound **3** where conditions 2 give the best selectivity.

The measured nucleoside and conjugate peaks of LC-MS/MS analysis were integrated and the resulting areas constitute the raw data. This raw data was then processed considering the above normalization parameters: (i) n_A:_ for intersample comparability, the raw data was normalized to the MS peak area of adenosine as the internal standard (ii). To compare the extent and ratio of the reactions, correction factors r_f_ based on UV absorption at 320 nm were established. The areas of all conjugate peaks were integrated in both chromatograms (320 nm and MS) and their ratio corresponds the r_f_ values in Table S1-S6 in File S1. Table S7 in File S1 gives an overview over correction thus determined. The sequential application of n_A_ and r_f_ factors to raw data leads to an unbiased dataset to compare the reactivity of the coumarins (Figure 3) (iii). The abundance of each nucleoside in the tRNA *E. coli* samples was calculated (see Table S8 in File S1) and the resulting c_ra_ values applied to relate the reactivity dataset to the amount of target nucleosides. With this an overview on the nucleoside selectivity was achieved (Figure 4).

## Results and Discussion

### Reaction products of 4-bromomethyl-7-methoxycoumarin (BMB) with tRNA

To reproduce the reported selectivity of BMB, we decided to recapitulate the experimental settings [36], using BMB in alkylation reactions of total tRNA from *E. coli*, which is known to contain at least 1 pseudouridine per tRNA. This condition set 1 is characterized by a slightly acidic pH of 6.5 and high content of DMSO as solvent (75%). As a negative control without modified nucleotides, an in-vitro-transcript (IVT) of *M.m.* tRNA^Asp^ was used [42]. After the reaction with BMB, the samples were precipitated to remove DMSO from the reaction mixture and analyzed on a polyacrylamid gel. The left panel of Figure 2A shows fluorescence of PAGE analysis upon excitation at 365 nm, monitored with a GelDoc. The fluorescence indicates that the *E. coli* tRNA is covalently attached to the coumarin BMB after the labeling reaction. The panel on the right side shows an equally fluorescent band for the reaction of BMB with the non-modified *in-vitro*-transcript. The staining control with GelRed indicates similar amounts of loaded tRNA on the gel which implies that both tRNAs have reacted with BMB to a similar extent. Since the *in-vitro-*transcript only contains the four major bases and no pseudouridine, it appears that BMB is not selective for pseudouridine under these conditions. Intriguingly, the fluorescence in both bands is comparable, which suggests that the main contribution of the reaction products with BMB comes from a canonical base, rather than from a modified nucleotide such as thiouridine or pseudouridine.

To determine which nucleotides actually reacted with BMB, the alkylated tRNA was digested to nucleosides. HPLC analysis was applied to separate the various coumarin-nucleoside-conjugates. The putative reaction of BMB with nucleosides, with uridine (U) as an example, is shown in Figure 2B. Figure 2D shows a complete digest of total tRNA *E. coli* treated with BMB and analyzed on an HPLC equipped with a diode array detector (DAD). The red chromatogram (monitoring 254 nm) shows the presence of the four major nucleosides. In the later part of the chromatogram the green curve monitoring the coumarin absorption maximum at λ=320 nm shows 5 peaks for possible BMB-nucleoside-conjugates.

After identification of the prominent masses in these 5 peaks by operating the mass spectrometer (MS) in scanning mode, the respective precursor masses were subjected to collision induced fragmentation (CID) and the MS set to product ion scan mode. Figure 2C shows an exemplary fragmentation of a BMB-conjugate thus identified as uridine-BMB (U-BMB). An overview over all found BMB-conjugates and their fragmentation patterns used in the subsequently applied multiple reaction monitoring (MRM) method can be found in Table S1 in File S1. With this method the major peaks from the green UV chromatogram of Figure 2D were identified as Ψ-BMB (R_t_=14.3 and 14.7), G-BMB (R_t_=13.5 and 16.0), U-BMB (R_t_=15.5 min) and s^4^U-BMB (R_t_=16.2 min) (Figure 2E, blue chromatogram). Eluting right after U-BMB is 4-hydroxylmethyl-7-methoxy-coumarin(R_t_=15.9 min) which is not shown in the MS/MS chromatogram and was not further examined as it is considered a side product that does not further interfere with the labeling reaction.

### Considerations for semi-quantitative analysis

There are three normalization caveats in the quantitative assessment of peak integrals from LC-MS/MS runs, for each of which a normalization factor was determined. These concern (i) the injected amount of sample for which the normalization factor n_A_ relates each peak area to that of the adenosine peak as internal standard. This normalization is displayed for BMB on the left side of Figure 3A. Here, the conjugate with uridine and guanosine (G) seem to be the most prominent reaction products. Furthermore (ii) differential detection efficiency by mass spectrometry is accounted for by the response factor r_f_ and displayed in the middle. Now, U-BMB appears as the major reaction product and comparison with the left graph reveals and overrepresentation of G-BMB due to an outstanding detection efficiency by the MS and therefore needs correction by r_f_. The determination of r_f_ is achieved by comparison of the MS signal with the UV signal of the conjugate. Akin to extinction coefficients for UV absorption, the response factor r_f_ reflects differential detection efficiency by MS, e.g. as a consequence of differential ionization efficiencies rooted in the chemical structure of the analytes. Of central importance, r_f_ values allow relative quantification for amounts of coumarin conjugate that are too low to produce UV signals. Since absolute values can only be obtained by using macroscopic amounts of conjugates as standards, we have determined relative values by comparison with UV absorption traces. Thus obtained response factors are based on the approximation that the coumarin moiety displays a comparable extinction coefficient in each conjugate. This implies that peaks with identical absorption at 320 nm originate from comparable amounts of coumarin conjugate. In that case, comparison of the MS signal of those same peaks is a measure of their relative detection efficiency by MS. Correspondingly, r_f_ values were extracted in multiple (n=3) runs by extracting quantitative correlation data of an MS signal to the corresponding coumarin signal at 320 nm in the UV chromatogram (Table S1 in File S1). The third correction factor concerns (iii) the relative abundance of nucleosides in the starting material RNA preparation, which is accounted for by application of c_ra_ ; a correction factor for relative abundance. The impact of c_ra_ for BMB is illustrated on the right of Figure 3A. Although U-BMB is the major reaction product, 4-thiouridine is the major reaction partner with the coumarin after consideration of general nucleoside abundance in the tRNA preparation. After application of all three correction factors it becomes apparent, that the selectivity of BMB towards pseudouridine is comparable to uridine, and factor 5 lower than for 4-thiouridine.

Because these findings are in clear contradiction to the previously published results [36], and because they do not offer a basis for selective labeling of minor or major nucleotides in RNA, we decided to explore if, in principle, bromomethylcoumarins can be developed as selective labeling agents by exploring structure-function relationships between substitution patterns on the coumarin ring and RNA alkylation.

### Structure-function relationship studies of 4-bromomethylcoumarins with RNA

A small panel of bromomethylcoumarins used in structure-function relationship studies is shown in Figure 1. These derivatives differ only in their substitution patterns at positions 6 and 7, which are remote to the reaction site. The choice of these compounds was therefore expected to reduce steric effects to a minimum, while differential mesomeric and inductive effects would affect electron density at the exocyclic bromomethylgroup as a key parameter for reactivity and selectivity. Compound **2** is a structural isomer to BMB with the methoxy-group attached to *C6* instead of *C7*; differential reactivity within this pair may arise from positional mesomeric effects as well as from inductive effects. A further pair, the constitutional isomeric methyl-substituted compounds **3** and **4**, was designed to deconvolute positional inductive effects only. A final pair used for this study comprised two phenyl-annulated coumarins (compounds **5** and **6**).

In a first step MRM detection methods for the conjugates of the 5 additional coumarins (see Table S2-S6 in File S1) were developed and their corresponding response factors r_f_ established as described for the BMB conjugates (again n=3). BMB and the 5 coumarins **2-6** were then reacted with total tRNA *E. coli* using the previously established condition set 1, and analyzed by LC-MS. The nucleoside composition of the tRNA remained essentially unaffected, indicating that depurination upon *N7* alkylation did not occur to a significant extend (Figure S2 in File S1). The same nucleotides guanosine, uridine, 4-thiouridine and pseudouridine were found to react with all coumarins.

The upper graph of Figure 3B shows the relative frequency of the detected conjugates after n_A_ and r_f_ correction. It is immediately apparent, that the substitution pattern of the coumarin has a significant influence on both, overall and relative reactivity. Compared to its isomer and the other coumarin derivatives, BMB is the most reactive compound, and the only one with a clear preference for uridine. In general it can be observed, that the *C7* substituted (or h-annulated, respectively) compounds show an overall higher reactivity than the *C6* substituted (f-annulated) counterparts, and that conjugates with uridine or 4-thiouridine are formed in roughly similar absolute amounts (i.e. prior to c_ra_ correction). One interesting exception is compound **3** which is mostly conjugated to 4-thiouridine. Correction of nucleoside abundance with factor c_ra_ reveals 4-thiouridine as the main reaction partner for all tested coumarins as can be seen in the upper row of Figure 3C. The comparison of the upper rows of Figure 3B and C confirms the outstanding behavior of compound **3** towards 4-thiouridine.

### Influence of the reaction conditions

A second set of reaction conditions was used to study the effect on nucleoside reactivity and selectivity. While reactant concentrations, DMSO content and temperature were kept constant, the buffer pH was elevated to more alkaline pH 8.25. An influence is immediately apparent when comparing the upper graph (conditions 1) of Figure 3B with the graph below (conditions 2). The obviously increased overall reactivity at alkaline pH is presumably a consequence of substrate deprotonation [44]. The increase is most prominent for uridine and surprisingly accompanied by an opposing, i.e. decreased reactivity towards guanosine. This is most obvious for BMB, but a similar trend applies to all other compounds. It is noteworthy that under reaction conditions 1, the early eluting G conjugate (G/1) is predominant, while under conditions 2 the later eluting conjugate G/2 is the main guanosine conjugate. Some interesting relationships of structure and function can be extracted from this data. For example, the 7 position of the methoxy-group on the coumarin, when compared to the 6 position, causes an increase of the overall reactivity in both condition sets. A less pronounced but similar effect of substituents of the 6 vs. 7 positions can be found within the other isomeric coumarin pairs, with the possible exception of compound **3** in condition set 2. Careful comparison of the upper and lower graph of Figure 3C, however, reveals a decrease in selectivity which is most prominent for BMB.

Figure 3B and C show in compliance with common principles of chemical selectivity, condition set 2 is associated with higher reactivity and lower selectivity alike for nearly all compounds, with the notable exception of compound **3**. This decrease in selectivity becomes more apparent in Figure 4 which is a display of comparative conjugate quantification.

The left graph of Figure 4 is a display of pseudouridine selectivity towards uridine. Although the least reactive compound **6** displays certain pseudouridine selectivity (~2 fold over uridine) neither of the tested conditions nor of the differentially substituted bromomethylcoumarin agents allows selective alkylation for pseudouridine to any significant extent. This is in some contrast to previously published data on BMB [36].

The selective labeling of thiouridines, reported by the same authors [18], could be well reproduced (right graph of Figure 4). Indeed, the most obvious feature thus revealed is the dominant reactivity of 4-thiouridine, which is easily rationalized by the nucleophilic properties of the sulfur [35]. The highest selectivity for 4-thiouridine, as defined by the ratio of the s^4^U-conjugate to the sum of the three others, is displayed by compound **3**, which reaches a value near 30.

## CONCLUSION AND OUTLOOK

A small panel of six bromomethylcoumarins was tested for reactivity and selectivity towards RNA nucleotides, including modified nucleotides present in *E. coli* tRNA under 2 sets of reaction conditions. Our previous study with the uridine selective coumarin N3BC revealed a complete loss of secondary and tertiary interactions of the target tRNA under the influence of 70% DMSO in the reaction mixture. We, therefore, expect the same complete accessibility of all major and modified nucleotides in the tRNAs used and no base-pairing effect should interfere with the alkylation reaction. Bromomethylcoumarin-conjugates with the four nucleotides uridine, guanosine, 4-thiouridine and pseudouridine were identified. Since the nucleophilic sites in urdine (*N3*) and 4-thiouridine (*S4*) are well characterized, it is not surprising to find a single conjugation product of each, uridine and 4-thiouridine. Pseudouridine and guanosine, however, have two and three free nitrogens, respectively, that are potential alkylation sites and can lead to multiple isomeric conjugates. Indeed, three different guanosine conjugates were observed under these reaction conditions, of which the most abundant one is presumably alkylated on the highly nucleophilic *N7* [43]. Only one major conjugate of pseudouridine is apparent. Previously unpublished data on N3BC alkylation support the *N3* alkylated pseudouridine conjugate as the supposed main product by comparing the pH dependence of the absorption spectra (See Figure S3 in File S1). As pseudouridine and guanosine display two and three alkylating sites, respectively, there is also the possibility of multiple alkylation of a single nucleoside. However, such conjugates were not observed after extensive scouring.

For quantification of coumarin-nucleoside conjugates, LC-MS/MS methods for each coumarin were developed. A comparison of the absolute amounts allowed assessing the overall reactivity (Figure 3B), while a representation of the same data normalized to nucleoside content of *E. coli* tRNA facilitates data interpretation in terms of selectivity (Figure 3C). The observed increase in reactivity upon shifting to more alkaline pH is in agreement with expectations. Effects on the site-specificity of guanosine alkylation were also observed. Positional effects of substituents on the aromatic systems show obvious influence on reactivity, although a general rationale as to the influence of mesomeric and inductive effects remains elusive. For example, the position of the methoxy-substituent in BMB and its isomer compound **2** has a pronounced effect on the coumarin reactivity. This may be related to changes in the electronic properties of the coumarin scaffold due to the +M effect of the methoxy groups. Such a positional effect was much less apparent for the methyl-substituted isomer pair **3** and **4**, which increase electron density only via a (short range) +I-effect of the methyl group. In terms of 4-thiouridine selectivity, the position of the methyl-group at *C7* appears most efficient and even changes in the reaction conditions do not significantly affect selectivity. In this respect, compound **3** is a clear exception, as the selectivity of all other compounds is strongly influenced by the reaction conditions (see Figure 4). As a lesson learned in efforts of selective labeling of a modified nucleoside, one should thoroughly optimize reaction conditions of RNA labeling procedures, as their influence is stronger than that of positional substituents.

Overall, our data clearly illustrates that relatively minor changes to structure and electronic properties of the coumarin scaffold do indeed significantly affect both reactivity and selectivity towards different nucleosides. Both, the position and the nature of the substituent are effective in this respect. However, although some trends are generally apparent, there are always exceptions. Interestingly, these exceptions mostly concern compound **3**, which incidentally also displays the highest selectivity for 4-thiouridine of all compounds. With the recently increasing attention to the biology of RNA modification [45,46], chemical tools are needed to label and detect modified nucleotides on a transcriptome wide scale, likely involving RNA Sequencing methods of the next generation [31–33]. Here, maximum selectivity will be the crucial factor to reduce false positives resulting from alternative site alkylation. Based on the present exploratory study, we predict that the coumarin scaffold can be used for achieving exquisite 4-thiouridine selectivity, using a similar substitution pattern as in coumarin **3** with improved electronical properties and slightly tuned reaction conditions.

## Supporting Information

File S1 Contains: Table S1: Retention times, mass transitions and correction factors of BMB nucleoside conjugates. Table S2: Retention times, mass transitions and correction factors of compound **2** nucleoside conjugates. Table S3: Retention times, mass transitions and correction factors of compound **3** nucleoside conjugates. Table S4: Retention times, mass transitions and correction factors of compound **4** nucleoside conjugates. Table S5: Retention times, mass transitions and correction factors of compound **5** nucleoside conjugates. Table S6: Retention times, mass transitions and correction factors of compound **6** nucleoside conjugates. Table S7: Overview of all correction factors and standard deviations. Table S8: Analysis of tRNA composition. Figure S1: Gel analysis of tRNA in-vitro-transcript (IVT) and tRNA *E. coli*. Figure S2: Major base composition is not altered upon coumarin treatment. Figure S3: UV spectrometrical changes in absorption by pseudouridine alkylation at different pH.
(DOCX)
