# Supplementary material for: Structure-Function Relationship of Substituted Bromomethylcoumarins in Nucleoside Specificity of RNA Alkylation
Source: PLoS One. 2013 Jul 3;8(7):e67945. doi: 10.1371/journal.pone.0067945 (PMC3700928; doi:10.1371/journal.pone.0067945)
Supplement: File S1 — Contains: Table S1: Retention times, mass transitions and correction factors of BMB nucleoside conjugates. Table S2: Retention times, mass transitions and correction factors of compound 2 nucleoside conjugates. Table S3: Retention times, mass transitions and correction factors of compound 3 nucleoside conjugates. Table S4: Retention times, mass transitions and correction factors of compound 4 nucleoside conjugates. Table S5: Retention times, mass transitions and correction factors of compound 5 nucleoside conjugates. Table S6: Retention times, mass transitions and correction factors of compound 6 nucleoside conjugates. Table S7: Overview of all correction factors and standard deviations. Table S8: Analysis of tRNA composition. Figure S1: Gel analysis of tRNA in-vitro-transcript (IVT) and tRNA E. coli. Figure S2: Major base composition is not altered upon coumarin treatment. Figure S3: UV spectrometrical changes in absorption by pseudouridine alkylation at different pH. (DOCX) [file pone.0067945.s001.docx]

Supporting information

Structure-function relationship of substituted bromomethylcoumarins in nucleoside specificity of RNA alkylation

Stefanie Kellner^[a]^, Laura Bettina Kollar^[a]^, Antonia Ochel^[a]^, Manjunath Ghate ^[b]^ & Mark Helm*^[a]^

^[a]^ Institute of Pharmacy and Biochemistry, Johannes Gutenberg University Mainz, Mainz, Germany

^[b]^ Institute of Pharmacy, Nirma University, Ahmedabad, Gujarat, India

* Phone: +49-6131-3925731. Fax: +49-6131-3920373. Email: mhelm@uni-mainz.de

Supplemental data:

Table S1: Retention times, mass transitions and correction factors of BMB nucleoside conjugates

Table S2: Retention times, mass transitions and correction factors of compound **2** nucleoside conjugates

Table S3: Retention times, mass transitions and correction factors of compound **3** nucleoside conjugates

Table S4: Retention times, mass transitions and correction factors of compound **4** nucleoside conjugates

Table S5: Retention times, mass transitions and correction factors of compound **5** nucleoside conjugates

Table S6: Retention times, mass transitions and correction factors of compound **6** nucleoside conjugates

Table S7: Overview of all correction factors and standard deviations

Table S8: Analysis of tRNA composition

Figure S1: Gel analysis of tRNA *in-vitro*-transcript (IVT) and tRNA *E. coli*.

Figure S2: Major base composition is not altered upon coumarin treatment.

Figure S3: UV spectrometrical changes in absorption by pseudouridine alkylation at different pH.

| Table S1: Retention times, mass transitions and correction factors of BMB nucleoside conjugates, established by measurements of calibration solutions | | | | | | | | |
| --- | --- | --- | --- | --- | --- | --- | --- | --- |
|  | R_t_  [min] | Mass transition |  | UV | MS/MS | c_f_ | Average c_f_ | Standard deviation |
| G-BMB 1 | 13.5 | 472.3  🡪 340.2 | # 1 | 14.3 | 283679 | 19852 | 22194 | 3313 |
|  |  |  | # 2 | 11.4 | 279714 | 24536 |  |  |
| G-BMB 2 | 16.0 | 472.3  🡪 340.2 | # 1 | 6.6 | 83044 | 12621 | 14961 | 3309 |
|  |  |  | # 2 | 4.9 | 84947 | 17301 |  |  |
| Ψ-BMB | 14.3 | 433.1  🡪 343.2 | # 1 | 18.6 | 10705 | 575 | 631 | 85 |
|  |  |  | # 2 | 18.2 | 10758 | 590 |  |  |
|  |  |  | # 3 | 21.3 | 15544 | 730 |  |  |
| U-BMB | 15.5 | 433.1  🡪301.2 | # 1 | 118.9 | 367643 | 3092 | 3079 | 52 |
|  |  |  | # 2 | 118.0 | 368685 | 3124 |  |  |
|  |  |  | # 3 | 225.6 | 681742 | 3022 |  |  |
| s^4^U-BMB | 16.2 | 449.2  🡪 317.2 | # 1 | 44.3 | 35197 | 794 | 824 | 43 |
|  |  |  | # 2 | 41.1 | 35093 | 854 |  |  |

| Table S2: Retention times, mass transitions and correction factors of compound **2** nucleoside conjugates, established by measurements of calibration solutions | | | | | | | | |
| --- | --- | --- | --- | --- | --- | --- | --- | --- |
|  | R_t_  [min] | Mass transition |  | UV | MS/MS | c_f_ | Average c_f_ | Standard deviation |
| G-**2** / 1 | 13.5 | 472.2  🡪 340.4 | # 1 | 2.5 | 180645 | 73733 | 54694 | 26925 |
|  |  |  | # 2 | 5.0 | 178274 | 35655 |  |  |
| G-**2** / 2 | 15.9 | 472.2  🡪 340.4 | # 1 | n.a. |  |  | As G-**2** / 1 | As G-**2** / 1 |
|  |  |  | # 2 | n.a. |  |  |  |  |
| Ψ-**2** | 14.3 | 433.0  🡪343.4 | # 1 | 2.2 | 5206 | 2366 | 2640 | 292 |
|  |  |  | # 2 | 2.0 | 5212 | 2606 |  |  |
|  |  |  | # 3 | 2.4 | 7132 | 2947 |  |  |
| U-**2** | 15.5 | 433.2  🡪 301.4 | # 1 | 24.2 | 227431 | 9410 | 8971 | 820 |
|  |  |  | # 2 | 24.0 | 227193 | 9478 |  |  |
|  |  |  | # 3 | 54.8 | 439622 | 8025 |  |  |
| s^4^U-**2** | 16.1 | 449.2  🡪 317.2 | # 1 | 22.0 | 55959 | 2551 | 2401 | 146 |
|  |  |  | # 2 | 19.0 | 45482 | 2394 |  |  |
|  |  |  | # 3 | 17.4 | 39427 | 2259 |  |  |

n.a.: peak too small for integration and partly co-elution with s^4^U-**2**

| Table S3: Retention times, mass transitions and correction factors of compound **3** nucleoside conjugates, established by measurements of calibration solutions | | | | | | | | |
| --- | --- | --- | --- | --- | --- | --- | --- | --- |
|  | R_t_  [min] | Mass transition |  | UV | MS/MS | c_f_ | Average c_f_ | Standard deviation |
| G-**3** / 1 | 14.0 | 456.2  🡪 324.4 | # 1 | 1.4 | 56134 | 39811 | 38515 | 1834 |
|  |  |  | # 2 | 1.5 | 56934 | 37218 |  |  |
| G-**3** / 2 | 16.3 | 456.2  🡪 324.4 | # 1 | 0.8 | 11664 | 14765 | 13705 | 1292 |
|  |  |  | # 2 | 0.8 | 11409 | 14085 |  |  |
|  |  |  | # 3 | 2.0 | 24777 | 12266 |  |  |
| Ψ-**3** | 14.7 | 417.2  🡪 327.4 | # 1 | 3.9 | 3466 | 891 | 875 | 26.5 |
|  |  |  | # 2 | 3.9 | 3446 | 890 |  |  |
|  |  |  | # 3 | 3.4 | 2864 | 845 |  |  |
| U-**3** | 15.9 | 417.2  🡪 285.3 | # 1 | 47.6 | 410224 | 8618 | 8410 | 335 |
|  |  |  | # 2 | 16.9 | 146191 | 8630 |  |  |
|  |  |  | # 3 | 17.5 | 148390 | 8475 |  |  |
|  |  |  | # 4 | 20.1 | 159403 | 7919 |  |  |
| s^4^U-**3** | 16.5 | 433.2  🡪 301.3 | # 1 | n.a. |  |  |  |  |
|  |  |  | # 2 |  |  |  |  |  |

n.a.: peak co-eluted with hydroxyl-compound **3**, no integration possible

| Table S4: Retention times, mass transitions and correction factors of compound **4** nucleoside conjugates, established by measurements of calibration solutions | | | | | | | | |
| --- | --- | --- | --- | --- | --- | --- | --- | --- |
|  | R_t_  [min] | Mass transition |  | UV | MS/MS | c_f_ | Average c_f_ | Standard deviation |
| G-**4** / 1 | 14.0 | 456.2  🡪 324.4 | # 1 | 3.4 | 186993 | 55488 | 55861 | 529 |
|  |  |  | # 2 | 3.2 | 178265 | 56235 |  |  |
| G-**4** / 2 | 16.3 | 456.2  🡪 324.4 | # 1 | 2.2 | 32077 | 14320 | 14920 | 848 |
|  |  |  | # 2 | 2.0 | 31349 | 15519 |  |  |
| Ψ-**4** | 14.7 | 417.2  🡪 327.4 | # 1 | 5.1 | 6470 | 1264 | 1025 | 272 |
|  |  |  | # 2 | 5.1 | 6406 | 1259 |  |  |
|  |  |  | # 3 | 3.0 | 2304 | 781 |  |  |
|  |  |  | # 4 | 2.9 | 2321 | 798 |  |  |
| U-**4** | 15.9 | 417.2  🡪 285.3 | # 1 | 23.5 | 317438 | 13489 | 11073 | 2395 |
|  |  |  | # 2 | 24.0 | 307332 | 12779 |  |  |
|  |  |  | # 3 | 40.0 | 363900 | 9102 |  |  |
|  |  |  | # 4 | 40.2 | 359249 | 8928 |  |  |
| s^4^U-**4** | 16.5 | 433.2  🡪 301.3 | # 1 | n.a. |  |  |  |  |
|  |  |  | # 2 |  |  |  |  |  |

n.a.: peak co-eluted with hydroxyl-compound **4**, no integration possible

| Table S5: Retention times, mass transitions and correction factors of compound **5** nucleoside conjugates, established by measurements of calibration solutions | | | | | | | | |
| --- | --- | --- | --- | --- | --- | --- | --- | --- |
|  | R_t_  [min] | Mass transition |  | UV | MS/MS | c_f_ | Average c_f_ | Standard deviation |
| G-**5** / 1 |  | 492.2  🡪 360.4 | # 1 | 3.5 | 115456 | 33466 | 33962 | 1470 |
|  |  |  | # 2 | 3.7 | 123129 | 33369 |  |  |
|  |  |  | # 3 | 4.3 | 140408 | 32882 |  |  |
| G-**5** / 2 |  | 492.2  🡪 360.4 | # 1 | 3.0 | 27622 | 9207 | 8101 | 1610 |
|  |  |  | # 2 | 2.2 | 19272 | 8840 |  |  |
| Ψ-**5** |  | 453.2  🡪 363.4 | # 1 | 5.1 | 5532 | 1093 | 1047 | 97 |
|  |  |  | # 2 | 5.2 | 5523 | 1068 |  |  |
|  |  |  | # 3 | 6.2 | 6963 | 1121 |  |  |
|  |  |  | # 4 | 6.4 | 5779 | 906 |  |  |
| U-**5** |  | 453.2  🡪 321.4 | # 1 | 36.7 | 189306 | 5155 | 4425 | 859 |
|  |  |  | # 2 | 38.7 | 190805 | 4928 |  |  |
|  |  |  | # 3 | 67.1 | 294476 | 4389 |  |  |
|  |  |  | # 4 | 66.5 | 214697 | 3230 |  |  |
| s^4^U-**5** |  | 469.2  🡪 337.2 | # 1 | 23.1 | 40483 | 1754 | 1615 | 272 |
|  |  |  | # 2 | 23.7 | 41171 | 1743 |  |  |
|  |  |  | # 3 | 15.5 | 27254 | 1764 |  |  |
|  |  |  | # 4 | 15.3 | 18491 | 1208 |  |  |

| Table S6: Retention times, mass transitions and correction factors of compound **6** nucleoside conjugates, established by measurements of calibration solutions | | | | | | | | |
| --- | --- | --- | --- | --- | --- | --- | --- | --- |
|  | R_t_  [min] | Mass transition |  | UV | MS/MS | c_f_ | Average c_f_ | Standard deviation |
| G-**6** / 1 | 15.1 | 492.2  🡪 360.4 | # 1 | 2.0 | 70792 | 35396 | 35840 | 629 |
|  |  |  | # 2 | 2.0 | 72570 | 36285 |  |  |
| G-**6** / 2 | 16.9 | 492.2  🡪 360.4 | # 1 | 7.1 | 51858 | 7335 | 6246 | 1539 |
|  |  |  | # 2 | 8.5 | 44047 | 5158 |  |  |
| Ψ-**6** | 15.6 | 453.2  🡪 363.4 | # 1 | 4.9 | 4473 | 924 | 996 | 55 |
|  |  |  | # 2 | 4.8 | 4733 | 990 |  |  |
|  |  |  | # 3 | 7.9 | 8290 | 1055 |  |  |
|  |  |  | # 4 | 8.2 | 8283 | 1016 |  |  |
| U-**6** | 16.6 | 453.2  🡪 321.4 | # 1 | 14.0 | 40511 | 2894 | 3217 | 325 |
|  |  |  | # 2 | 15.0 | 45230 | 3015 |  |  |
|  |  |  | # 3 | 30.3 | 101609 | 3350 |  |  |
|  |  |  | # 4 | 27.1 | 97766 | 3610 |  |  |
| s^4^U-**6** | 17.1 | 469.2  🡪 337.2 | # 1 | 18.3 | 44814 | 2454 | 2168 | 390 |
|  |  |  | # 2 | 9.8 | 25024 | 2543 |  |  |
|  |  |  | # 3 | 19.2 | 36977 | 1913 |  |  |
|  |  |  | # 4 | 9.5 | 16519 | 1746 |  |  |

| Table S7: Overview of all correction factors and standard deviations | | | | | | | | | | | | | |
| --- | --- | --- | --- | --- | --- | --- | --- | --- | --- | --- | --- | --- | --- |
|  | 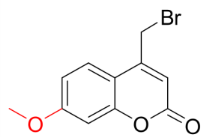 | | 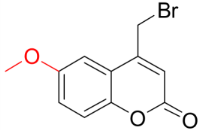 | | 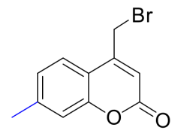 | | 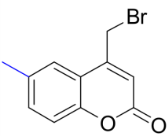 | | 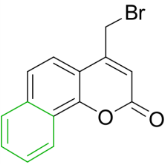 | | 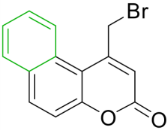 | | |
|  | BMB | | **2** | | **3** | | **4** | | **5** | | **6** | | |
| conjugate | Average | σ | Average | σ | Average | σ | Average | σ | Average | σ | | Average | σ |
| G / 1 | 22194 | 3313 | 54694 | 26925 | 38514 | 1834 | 55861 | 529 | 33962 | 1470 | | 35841 | 629 |
| G / 2 | 14961 | 3309 | as G /1 | as G /1 | 13705 | 1292 | 14920 | 848 | 8101 | 1610 | | 6246 | 1540 |
| s^4^U | 1027 | 353 | 2401 | 146 | 1321* | 294* | 2285^#^ | 116^#^ | 1615 | 272 | | 2169 | 390 |
| U | 3079 | 52 | 8971 | 820 | 8410 | 335 | 11073 | 2395 | 4425 | 859 | | 3217 | 325 |
| Ψ | 631 | 85 | 2640 | 292 | 875 | 26 | 1025 | 272 | 1047 | 97 | | 996 | 55 |

* calculated as average with corresponding correction factors of BMB and compound **5**

^#^ calculated as average with corresponding correction factors of compound **2** and compound **6**

| Table S8 determination of nucleoside composition of total tRNA *E.coli* | | | | | |
| --- | --- | --- | --- | --- | --- |
| Name | λ_max_ | ε_max_ (x10^-3^) | Area (λ_max_) | mol. frequency  Area (λ_max_) / ε_max_ | Nucleoside abundance |
| G | 253 nm | 13.6 | 284 | 20912 | 24.4 |
| s^4^U | 331 nm | 21.2 | 2.6 | 124 | 0.14 |
| U | 262 nm | 10.1 | 8.7 | 8616 | 10 |
| Ψ | 263 nm | 8.1 | 8.3 | 1027 | 1.06 |

$$tRNA composition= \frac{mol. frequency (nucleoside)}{mol. frequency (uridine)}\times10$$

To perform statements of selectivity, the analysis must also take into account that uridine and guanosine are more frequent in the substrate tRNA than the modified uridine residues s^4^U and Ψ. The composition of total tRNA *E.coli* was accessed by digesting untreated tRNA followed by LC-UV analysis and using the absorption of each nucleoside at its λ_max_ and division by its corresponding molar extinction coefficient known from literature [1].The data of tRNA composition is then used to equalize the adjusted areas to the nucleoside abundance, which finally reveals the favorite reaction partner of the coumarin (Figure 3).

**
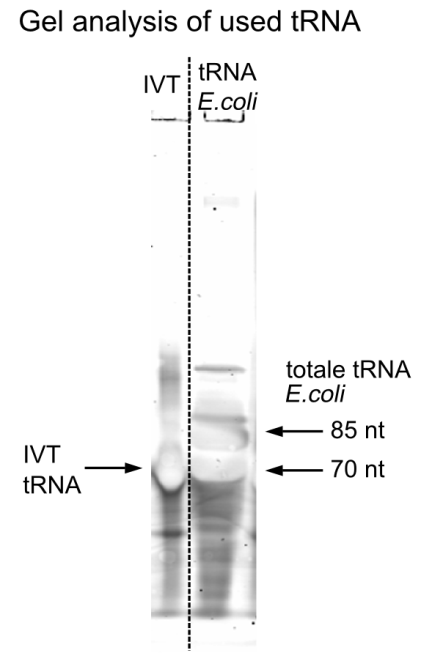

Figure S1.** Gel analysis of 50 µg tRNA *in-vitro*-transcript (IVT) and tRNA *E. coli*. Staining was performed with GelRed. Note: Due to the high concentration of tRNA, GelRed does not reach the inside of bands.Therefore, the highly concentrated tRNA band appears white.

**
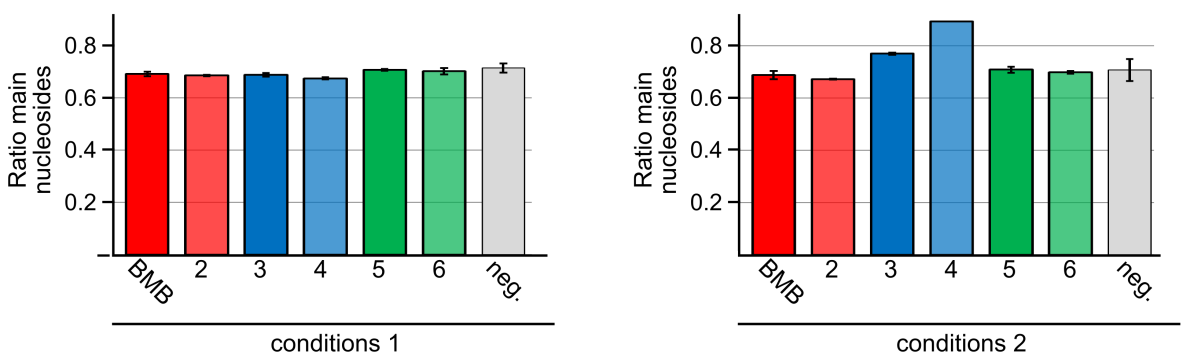
**

**Figure S2.** tRNA major base composition is not altered upon coumarin treatment. A) LC-MS analysis of tRNA treated under conditions 1. B) LC-MS analysis for conditions 2.


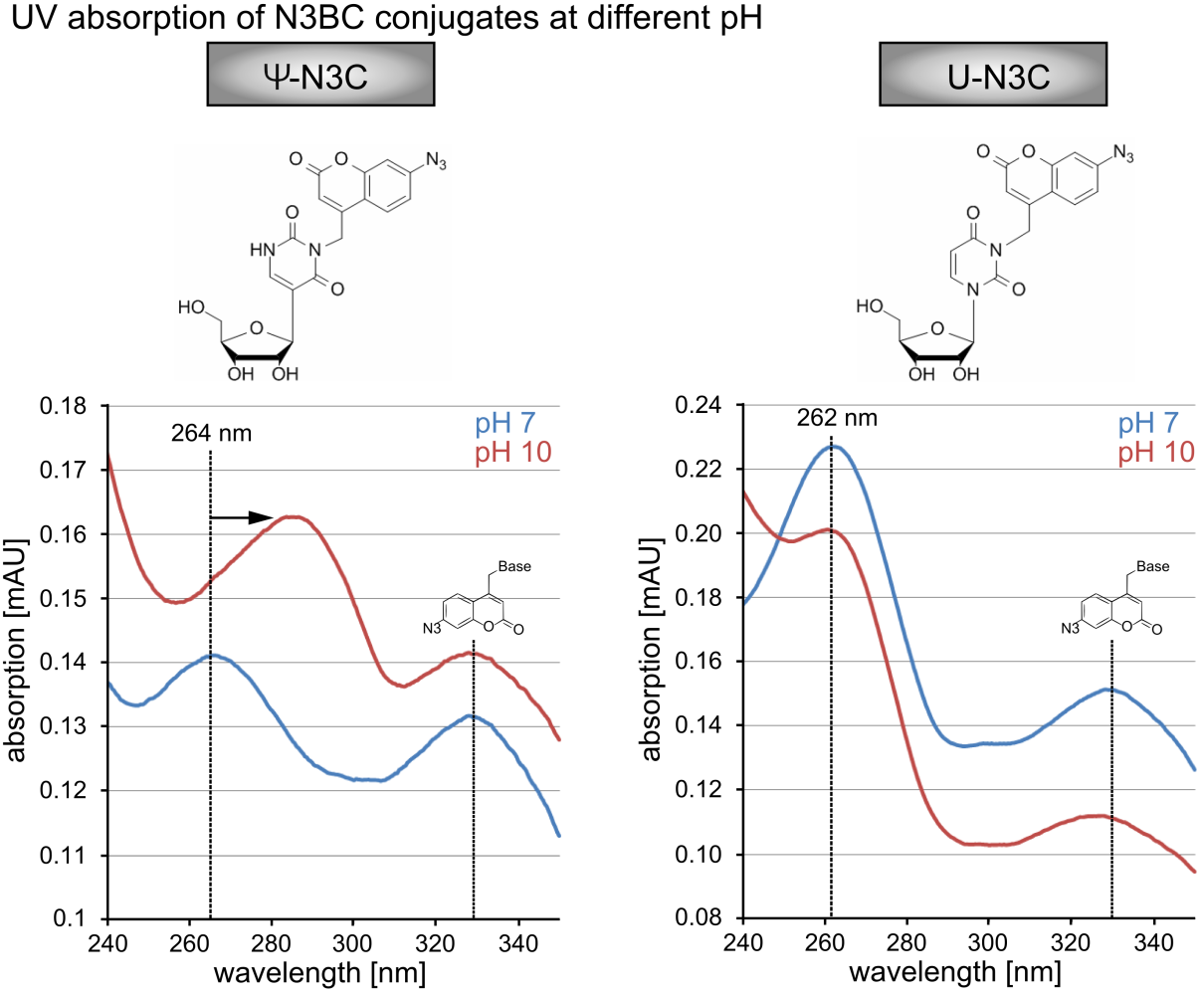


**Figure S3:** UV spectrometrical changes in absorption by pseudouridine alkylation at different pH. Isolated reaction products of pseudouridine (right) and uridine (left) with N3BC were examined as described by Ho and Gilham in 1971 for alkylation of pseuoduridine with CMCT [[2](#_ENREF_1)]. Since the early 1960 it was known that the UV absorption spectrum of pseudouridine at pH 10 is bathochromically shifted if the *N3* position is methylated. Photometrical examination of isolated 3-CMC-Ψ at pH 7 showed a λmax of 263 nm and pH 10 a λmax of 293 nm whereas the 1-CMC-Ψ had a λmax of 263 nm in both cases. Isolated Ψ-N3BC shows the same bathochromic shift as 3-CMC-Ψ at pH 10 (right) but U-N3BC has the same absorption maximum in both cases. We therefore conclude that the coumarin N3BC is mainly reacting with *N3* of pseudouridine and only to a minor extent with *N1*. Since the reaction site of the nucleophile is not influenced by the substitution pattern we conclude that all coumarins tested alkylate the *N3* position.

**References:**

1. Hall DBDaRH (May 2010) Purines, Pyrimidines, Nucleosides, and Nucleotides Handbook of Biochemistry and Molecular Biology. Fourth Edition ed. pp. 269-358.

2. Ho NW, Gilham PT (1971) Reaction of pseudouridine and inosine with N-cyclohexyl-N'-

beta-(4-methylmorpholinium)ethylcarbodiimide. Biochemistry 10: 3651-3657.
